# The implementation of health promotion in primary and community care: a qualitative analysis of the ‘Prescribe Vida Saludable’ strategy

**DOI:** 10.1186/s12875-017-0584-6

**Published:** 2017-02-17

**Authors:** Catalina Martinez, Gonzalo Bacigalupe, Josep M. Cortada, Gonzalo Grandes, Alvaro Sanchez, Haizea Pombo, Paola Bully, on behalf of the PVS group

**Affiliations:** 1 Primary Care Research Unit of Bizkaia, Basque Healthcare Service – Osakidetza, BioCruces Health Research Institute, Luis Power 18 4th floor, Bilbao, 48014 Spain; 2 University of Massachusetts Boston, College of Education and Human Development, 100 Morrissey Bvld, Boston, MA 02125 USA; 3 Deusto Primary Health Care Center. Bilbao-Basurto Integrated Care Organisation. Basque Healthcare Service – Osakidetza. BioCruces Health Research Institute, Luis Power 18, Bilbao, 48014 Spain

**Keywords:** Implementation Research, Program Evaluation, Qualitative Research, Organizational Innovation, Primary Health Care, Community Health Services, Health Promotion, Complex Interventions, Pilot Implementation

## Abstract

**Background:**

The impact of lifestyle on health is undeniable and effective healthy lifestyle promotion interventions do exist. However, this is not a fundamental part of routine primary care clinical practice. We describe factors that determine changes in performance of primary health care centers involved in piloting the health promotion innovation ‘Prescribe Vida Saludable’ (PVS) phase II.

**Methods:**

We engaged four primary health care centers of the Basque Healthcare Service in an action research project aimed at changing preventive health practices. Prescribe Healthy Life (PVS from the Spanish “Prescribe Vida Saludable) is focused on designing, planning, implementing and evaluating innovative programs to promote multiple healthy habits, feasible to be performed in routine primary health care conditions. After 2 years of piloting, centers were categorized as having high, medium, or low implementation effectiveness. We completed qualitative inductive and deductive analysis of five focus groups with the staff of the centers. Themes generated through consensual grounded qualitative analysis were compared between centers to identify the dimensions that explain the variation in actual implementation of PVS, and retrospectively organized and assessed against the Consolidated Framework for Implementation Research (CFIR).

**Results:**

Of the 36 CFIR constructs, 11 were directly related to the level of implementation performance: *intervention source, evidence strength and quality, adaptability, design quality and packaging, tension for change, learning climate, self-efficacy, planning, champions, executing,* and *reflecting and evaluating,* with *—organizational tracking* added as a new sub-construct. Additionally, another seven constructs emerged in the participants’ discourse but were not related to center performance: *relative advantage, complexity, patients’ needs and resources, external policy and incentives, structural characteristics, available resources*, and *formally appointed internal implementation leaders*. Our findings indicate that the success of the implementation seems to be associated with the following components: the context, the implementation process, and the collaborative modelling.

**Conclusions:**

Identifying barriers and enablers is useful for designing implementation strategies for health promotion in primary health care centers that are essential for innovation success. An implementation model is proposed to highlight the relationships between the CFIR constructs in the context of health promotion in primary care.

**Electronic supplementary material:**

The online version of this article (doi:10.1186/s12875-017-0584-6) contains supplementary material, which is available to authorized users.

## Background

The impact of health behaviors and lifestyles on health outcomes is undisputed [1] and its individual, social, environmental and cultural determinants are well known [2]. Primary health care (PHC) professionals have many opportunities to promote healthy behaviors in patients with effective interventions [3, 4]. However, healthy lifestyle promotion is far from being integrated in routine primary care practice [5–7]. Our own research group has contributed to generate evidence on the effectiveness of clinical interventions for health promotion through several clinical trials [8, 9]. Nevertheless, the delivery of primary care interventions tends to stop after PHC professionals participate in such trials [10]. This lack of integration of evidence-based interventions may be explained by weaknesses in implementation strategies [11].

Implementation strategies can be defined as sets of methods, techniques, and interventions used to enhance the adoption and integration of evidence-based innovations into usual care [12]. In order to build strong implementation strategies, we need to identify factors that determine change in practice, namely, barriers and enablers of change [13]. This requires learning from the experiences of all innovation agents. However, when implementing a new intervention or practice most of the people that can participate in such research have little or no experience of change in practice and, instead of reporting determinants of change, they report what determines their current performance. In the ‘Prescribe Vida Saludable’ (PVS) innovative project for healthy promotion by PHC professionals we had the opportunity to explore the experiences of individuals who were actively involved in changing clinical practice in health promotion.

The aim of this inquiry is to identify core factors associated with success and barriers in the implementation of the PVS intervention and assess factors associated with better performance in its piloting phase. We describe the experience of the PHC professionals who have already been involved in innovation and how they assess the successes and challenges of the implementation of PVS. The analysis is based on Damschroder et al.’s Consolidated Framework for Implementation Research (CFIR) [14] which is outlined in the methods section as part of the analytical strategy. Identifying core constructs associated with implementation should strengthen future dissemination and inform the modeling process of sound and effective implementation strategies for PHC professionals practice redesign.

## Methods

A qualitative comparative analysis design [15, 16] was used to comprehensively explore PVS implementation barriers and enablers. In addition to a comparative analysis of ongoing process indicators for each primary health care (PHC) center, the qualitative evaluation consisted of focus groups with PHC professionals involved. The study protocol was approved by the Primary Care Research Committee of the Basque Healthcare Services and by the Basque Country Clinical Research Ethics Committee.

### Participants

The four participating PHC centers were a convenience sample, selected by the medical directors of the primary care districts of the Basque Healthcare Service on the basis of their previous involvement in health promotion programs or preventive practice optimization initiatives. The PHC professional teams initiated an action research process to design the local PVS implementation strategy for each center. In brief, a bottom-up decision making processes was initiated in the four participating centers to select actions to be included in the implementation strategy, based on discussion and consensus meetings among a multi-professional primary care team and community members. We refer to this method as collaborative modeling facilitated by the research team. A coordinator at each center was the liaison with the research team and leaded the process at the local level. The Department of Public Health supported the teams, with a district public health department representative attending the aforementioned monthly meetings.

The PVS project emerged as an initiative facilitated by the Primary Care Research Unit of Bizkaia (UIAPB) of the Basque Health Service (Osakidetza). The research team provided external facilitation for changing clinical practice. Other participants in the project were community-based organizations, including nine local municipality departments, six schools, four sports facilities, and two manufacturing companies, as well as local councils and senior management of several Osakidetza departments (information technology, primary care, and public health).

### PVS innovation

**Fig. 1:**
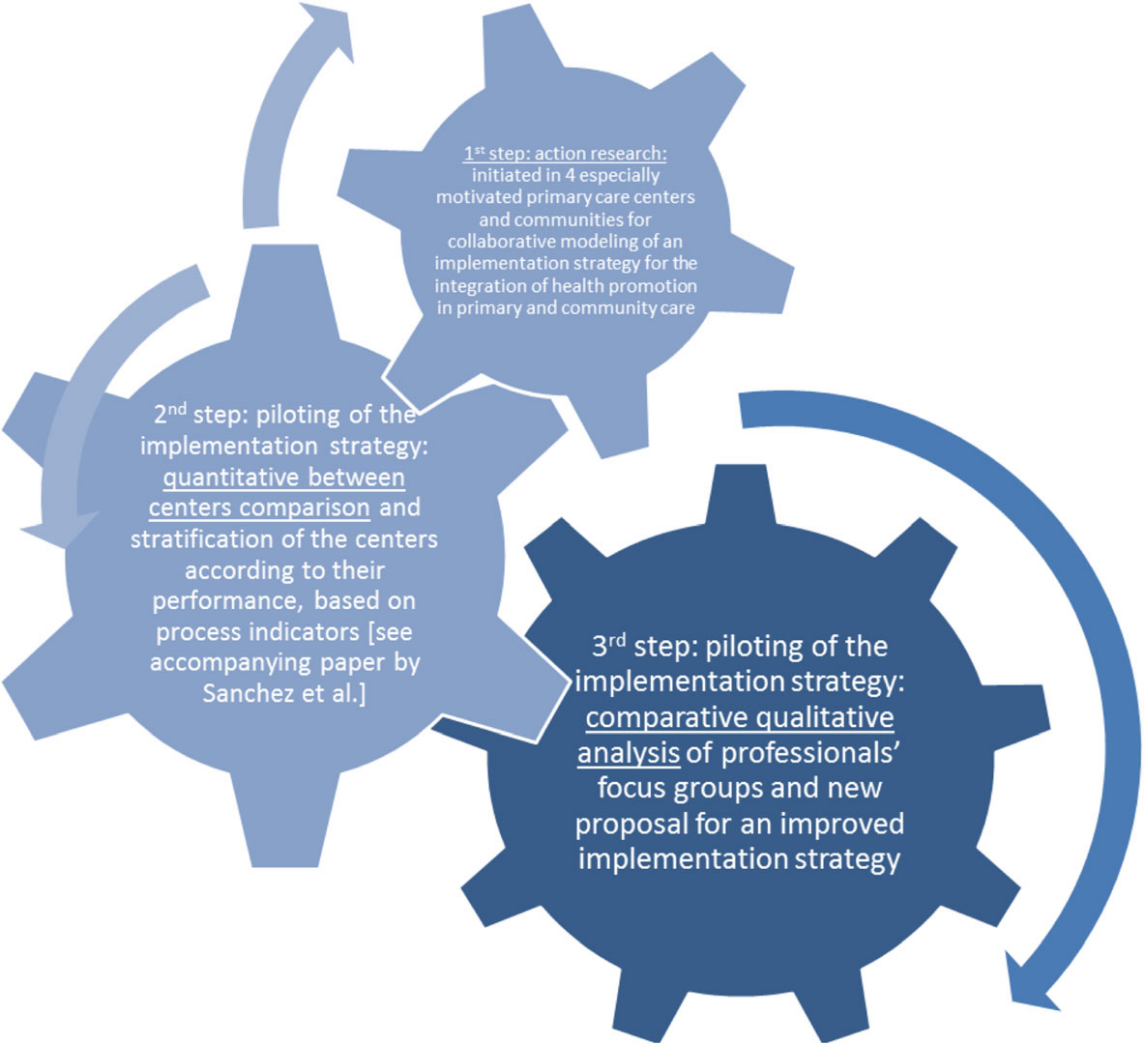
PVS research steps

The PVS first phase consisted of a collaborative modeling process to adapt evidence-based health promotion interventions to the specific contexts of the PHC centers and communities, and simultaneously change PHC professional organization (see Fig. 1). Most of the staff (80%) of the four participating PHC centers were actively involved in this process and they selected the 5 A’s evidence-based clinical intervention (A1: Assess, A2: Advise, A3: Agree, A4: Assist, and A5: Arrange Follow-Up) to address three healthy lifestyles: healthy diet, physical activity, and smoking cessation [3, 4, 17]. The second phase of PVS was the pilot implementation to evaluate the feasibility of the strategy. This pilot study included all 22,459 patients 10 to 65 years old who had attended a healthcare appointment over the 2-year study period. They were assessed for physical activity, diet, and smoking in 52% of cases, 33% of them received advice at least once for changing their behavior, and 10% were prescribed lifestyle changes, with a personalized plan (see accompanying paper by Sanchez et al.).

The Primary Care Research Unit provided PHC centers with a monthly progress report outlining the results of their activities, which included indicators of assessment of habits (A1), counseling and advising (A2), and prescription of changes in behavior with tailored plans (A4). These monthly reports were a cornerstone of the audit and feedback strategy. The research team met monthly with the coordinators of the healthcare teams to evaluate the program, discuss the implementation indicators, identify ways of increasing the effectiveness of the intervention, and overall reflect on what the teams were learning about the implementation process. The coordinators organized monthly meetings in their PHC centers with the same implementation improvement objectives.

### Implementation strategy

The PVS implementation strategy was focused on various levels: community involvement, top-down support from managers, bottom-up primary care organizational change, and the development of innovative e-health information and communication technologies. The study protocol has been published previously [18]. The participants adapted the 5As’ strategy into a community health center’s functioning plus at least one collaborative partner in the community through a collaborative modeling process. The research team facilitated a bottom-up decision making process, based on 8–10 discussion and consensus meetings among a multi-professional primary care team and community members. The intervention components did not all reside in the community health centers, A1 (Ask), for instance, could be accomplished at a school, a workplace, a PVS website link, or administrative personnel at the health center in advance of the primary care consultation. A2 (Advise) and A3 (Agree) were tasks mostly assigned to the family physicians, while the A4 (Assist) was often a role accomplished by nurses who prescribed and helped plan for health behavior modification. All the healthcare center professionals were involved in A5 (Arrange Follow Up) with client services playing an important role too.

New e-health tools were designed, tested and incorporated into the electronic health record (EHR). Screening for healthy behaviors was performed with paper-based self-report questionnaires, entered into the EHR by administrative staff, and with an electronic version publically available on the Basque Healthcare Service web portal for individual self-assessment, with online transmission of data to the EHR through a secure developed web service (see accompanying paper by Sanchez et al.). This screening provided automatic feedback to individuals and generated reminders in the EHR that prompted clinicians to implement health promotion activities guided by the PVS application integrated in the EHR information system. These reminders helped primary care staff to easily identify individuals whose lifestyles had not been assessed; to assess physical activity, diet, and smoking status of attendees; to identify those not meeting recommendations; and to select high priority populations for interventions based on data recorded in the EHR. Further, the PVS application guided clinicians in providing individually tailored advice based on effective communication of risks and benefits associated with lifestyle. It also facilitated the prescribing of plans for lifestyle modification, providing algorithms, evidence-based support, and recommendations, warnings, timetables and other information about community resources. Besides it facilitated monitoring of patients over a follow-up period. The PVS application integrated all the information, and made it available to all PHC center staff, thereby making it easier to track patients.

The fidelity and maintenance of the strategy was based on a monthly audit and feedback meetings, where indicators on patients’ results were analyzed.

### Implementation performance

Considering the 2-year process indicators of the PVS second phase pilot study, participating centers were categorized as having high, medium, or low implementation performance, based on their adoption and implementation of the PVS activities (see Fig. 1). One of the centers was categorized as having high and one low implementation performance, while two were assigned to the “medium” category. Another paper [see accompanying paper by Sanchez et al.] and Table 1 provide further details of the PVS intervention during the 2-year pilot implementation process.

**Table 1:** Characteristics and implementation indicators of the primary care centers participating in this PVS pilot

		Center A	Center B	Center C	Center D
Population size		6004	5509	9915	21621
Age target in the PVS intervention, years		10–65	10–65	10–65	>10
Participation by professionals Participates/total in centre (%)	Family physicians	5/5	4/5	5/5	10/11
	Nurses	5/5	4/5	8/8	6/13
	Admission staff	5/5	3/4	5/6	5/6
	Pediatricians	1/1	0/1	2/2	0/2
	Midwives	1/1		1/1	0/2
	Others	1/1			
	Total	18/18 (100%)	11/15 (73%)	21/22 (95%)	21/34 (61%)
Community agents	Municipalities	4	2	2	1
	Schools	2	3	2	0
	Sport facilities	1	1	1	1
	Businesses	3	1	0	2
	Pharmacies	1	0	0	1
	Associations and other agencies	0	4	0	1
Two-year implementation performance indicators					
A1: % attendees assessed for physical activity, diet and smoking	3009/3635 (82.8%) (81.5–84.0%)	1640/2568 (63.7%) (62.0–65.7%)	3209/5883 (54.5%) (53.3–55.8%)	3792/10373 (36.6%) (35.6–37.5%)	
A2: % of attendees advised on increasing physical activity, improving diet or smoking cessation	2136 (58.7%) (57.2–60.4%)	809 (31.5%) (29.7–33.3%)	2170 (36.9%) (35.6–38.1%)	2318 (22.3%) (21.5–23.1%)	
A4: % of attendees prescribed a behavior change plan	552 (15.24%) (14.0–16.3%)	377 (14.7%) (13.3–16.1%)	406 (6.9%) (6.2–7.5%)	840 (8.1%) (7.6–8.6%)	

### Qualitative data collection

Staff of each of the four PHC centers were invited to participate in five focus groups (the largest center requiring two groups) and these were completed over 5 weeks (see Fig. 1). Specifically, the coordinators of the participating centers were asked to invite all the PVS implementation participants, and 75 physicians, nurses, and administrative personnel participated. An average of 15 participants attended each group (SD = 3.54). The potential pool of participants was 109 ($\overline{\mathrm{X}}$ = 21.8; SD = 4.49). Participants consent to their contribution to be published.

One researcher facilitated all five focus groups at the PHC centers. The research team selected this type of group interview, since one of the objectives was that all participants of each center participate jointly, in order to grasp the point of view of each individual within the group. The interviewer was selected ensuring that she had no previous direct relationship with or knowledge of participants. The discussion was guided by a set of open-ended questions to identify what factors were associated with the degree to which the PVS program had been adopted: *What are your thoughts regarding the implementation of PVS in this health center? What has been the impact of PVS on daily work? How do you interpret the indicators (participants being shown charts and graphs)? What are the factors that explain the wide variation in implementation of PVS between centers and between different members of staff within each center? What is your assessment of the way how patients are being reached? What may cause problems in the implementation and can you suggest any solutions? What are the barriers to implementation of the PVS?*

Data were shared during the interview on indicators for the sixth month of the project, from the monthly report. These data provided a clear picture of three cases depending on the degrees of success in the implementation. An observer, a representative of the public health department in the PVS efforts, accompanied the facilitator in the interviews and wrote an ethnographic report with her own analysis of the focus groups. The data analysis was ongoing, iterative, and informed by the research team’s observations. The interviews were audio-recorded and transcribed for analysis.

### Data analysis

A consensual qualitative research approach comprising two data analysis strategies was used to guide the systematic analysis of the barriers and enablers influencing the implementation. The two strategies included inductive and deductive data analysis to ensure not only triangulation of the data sources but also their trustworthiness.

The inductive methodology consisted of basic grounded thematic analysis. Basic themes were extracted from the transcripts analyzed separately by research team members and then consensually validated in team meetings. The ethnographic notes prepared by the observers of the group interviews also aided this thematic analysis and helped the organization of the transcribed material.

On completion of the first thematic analysis, the research team concluded that deductive analysis utilizing the CFIR model could be useful to address the wealth of data and to focus on the task of assessing implementation dimensions. This coding framework was chosen because it offers a complete taxonomy of operationally defined constructs that can influence the adoption of complex programs. CFIR constructs are organized into five major domains [15]: (1) the characteristics of the intervention, (2) the outer setting including patients’ needs and resources, (3) the inner setting (i.e., how compatible the program is with existing interventions), (4) the process employed to implement the program, and (5) the characteristics of the individuals involved.

Each transcript was treated as a case and two members of the research team separately completed exhaustive within-case coding. To reach consensus, the team carried out reviews of each focus group. This within-case analysis was completed for each group and audits of the analysis were completed to ensure consistency. After completion of the within-case analysis, common themes across cases were identified and a rating value (valence) assigned to each code. These data reflect the attributes the participants associated with each of these concepts. We used the same criteria as Damschroder and Lowery [15] for assigning these ratings. Valences from +2 to −2 reflect a positive or negative influence of each construct on the organization, work processes, and/or implementation efforts [15]. The rating was completed with the same consensual data analysis as that used in the coding of the transcripts. The analysis required continuous comparison across groups, and the examination of patterns in the data. One of the researchers performed the analysis without knowing the quantitative outcomes of the health center in question to enhance the trustworthiness of the qualitative data analysis. At the end of the analysis, vignettes were selected to exemplify the CFIR dimensions and to highlight their association with the actual PVS outcomes at each center. The research team shared the analysis with the coordinators of the PHC centers to feedback the findings.

## Results

### What factors are associated with community health promotion and primary care innovation?

In our study, 18 of the 36 CFIR constructs proposed by Damschroder and analyzed by the research team present high levels of trustworthiness. According to the positive (+2, +1), neutral (0), or negative (−1, −2) valences assigned by participating staff, these constructs can be divided in two groups. One group includes 11 constructs associated with the actual level of success in the implementation performance; there is a trend across centers in the valences assigned by participants and this is correlated with the level of success or failure in implementation performance. The other set of seven constructs appears to be unrelated to the actual level of implementation performance (See Table 2).

**Table 2:** CFIR constructs associated with actual implementation performance

PVS Cases CFIR Constructs		Centers by level of implementation				
		High: Center A	Medium: Centers B and C		Low: Center D	
I. Intervention characteristics						
A. Intervention source		+2	+1	0	0	+ association
B. Evidence Strength & Quality		+2	+1	+1	−1	+ association
C. Relative advantage		+1	+1	+2	+1	Not associated
D. Adaptability		−2	0	−1	0	- + associated ^a^
F. Complexity		−2	−1	−1	−2	Not associated
G. Design Quality & Packaging		−1	−1	+1	+1	+ association^a^
II. Outer setting						
A. Patient Needs & Resources		−1	+1	−2	−1	Not associated
D. External Policy & Incentives		−2	−1	−2	−2	Not associated
III. Inner setting						
A. Structural Characteristics		−2	−2	−2	−2	Not associated
D. Implementation climate	1. Tension for Change	+1	0	+2	−1	+ association
	6. Learning Climate	+2	0	X	−1	+ association
E. Readiness for Implementation	2. Available Resources	−2	−1	−1	−1	Not associated
IV. Characteristics of individuals						
B. Self-efficacy		+2	−1	+1	−2	+ association
V. PROCESS						
A Planning		0	0	0	−2	+ association
B Engaging	2. Formally appointed internal implementation leaders	+2	+2	+2	+1	Not associated
	3. Champions	+1	+2	X	X	+ association
C Executing		+1	0	−1	−2	+ association
D Reflecting & Evaluating		+1	+1	−1	−1	+ association
Organizational Tracking		−2	0	−2	X	- association

^a^ There is a negative association with the lack of adaptability and with problems in the design and packaging of the intervention

For the other 18 CFIR constructs, insufficient data emerged to assess their potential association with performance. They can be considered less important for designing strategies for change in public PHC professional services. Despite the lack of data that emerged on these dimensions, they should be studied further to assess their meaning in relation to predicting implementation performance of healthy lifestyle interventions. For example, the lack of engagement of *external change agents* appeared only in one of the centers, the one which showed the highest implementation rating, and it appeared to be negatively valued (see Table 2).

Tables 3 and 4 include quotes that exemplify the dimensions that were or were not associated with the implementation performance. The valences assigned to the set of quotes are also included in each cell. The CFIR constructs not associated with the level of performance explain important barriers to and enablers of the implementation in general, but comparing the positive and negative values assigned to them by staff does not show a relation to the actual implementation observed in each center. They include: *relative advantage, complexity, patients’ needs and resources, external policy and incentives, structural characteristics, available resources,* and *formally appointed internal implementation leaders.* The CFIR constructs that distinguished between health teams with low, medium, and high implementation performance included: *intervention source, evidence strength and quality, design quality and packaging, adaptability, tension for change, learning climate, self-efficacy, champions, reflecting and evaluating, planning,* and *executing*.

**Table 3:** CFIR constructs associated with PVS performance

CFIR Constructs	Cases		
	High implementation: Center A	Medium implementation: centers B and C	Low implementation: center D
Intervention characteristics			
Intervention source	*It was a collaborative effort at all levels; everyone had internalized their commitment to health promotion.* (+2)	*We have adhered voluntarily and we have joined because we want to respond to the challenge* (of making prevention effective in increasing healthy habits). (+1)	*This is something that we initially did voluntarily but then all sorts of demands were placed on us at the same time. (*0)
Evidence strength and quality	*We are seeing how our patients are changing their habits, similar to how we have seen a change in our own.* (+2)	*This is a priority, in other words, it is so important because of the impact it has. (*+1) *Coming from pediatrics, I think other types of things could be done to make things better because this tool doesn't fit very well.* (+1)	*Everyone as much as they can (…). Haven’t they told you that many folks say that five fruit or veg. portions can make you fat?* (−1)
Adaptability	*The method, in itself, is not bad. The problem is how fast. They are demanding a 95% and we are going at full speed, we can’t keep up with that for a long time.* (−2)	*For this outcome data to be useful, I would have to reach 4 or 5 daily, with each consultation being half an hour, there is not enough time for me and I create a waiting list for other demands.* (0)	*The surveys are a little complicated for people, aren’t they? About the portions and having to write it down every time, and all that; I think it doesn't reflect reality very well.* (−2)
Design quality & packaging	*(…) we thought, “that’s fantastic, we are going to have a tool that’s going to help us, and not the other way around, …” and gives us many, too many problems* (−1) *You’re working with a tool that you know doesn't work; we are even having trouble printing the prescriptions* (−1) *The tools didn’t work from the start, not theirs* (the nurses) *or ours* (admission)*; I’m talking about the assessment too (−1)*	*We should have better things that can help us, with this, I don't know where to fit it in* (−1) *The computer platform is amazing as a model* (…) *But when you want to give advice, you cannot print; it often fails completely or doesn’t work properly and it could be improved. I guess… 80% of my expectations are fulfilled while 20% aren’t because there are a lot of things that could be improved.* *We have to be positive, I think the tool we have is very good. In the long term, it is going to reap results (…) but it is a question of years.* (+1)	*For instance, physical exercise: I have always prescribed physical exercise; but to have the tool, the information sheets, the specifics and ways of telling individuals how much to do, in what ways, and how to orient patients about physical exercise.* (+2)
Inner setting: Implementation climate			
Tension for change	*We have committed to this and have internalized that it is not only about addressing the pathology but also about promoting health across the various levels of care.* (+1)	*We have to be positive and I think the tool is terrific. I think, in the long term, we will have good results.* (+2)	*I do tobacco prevention too (…) indeed this year, I have two or three of my patients have quit smoking; I have not managed the problem well because I don't feel comfortable with the program. I have not done the program with the patients.* (−2)
Learning climate	*All the time, we are trying to do more because everyone is saying ‘let’s do it, let’s do it’.* (+2)	*We have to do it gradually, without stressing, and always making it better.* (0) *I haven’t got as involved as him.* (0)	*I joined in December and until now I have not done anything.* (−1)
Characteristics of individuals			
Self-efficacy	*This is part of an effort on the part of the whole group and totally a personal effort, without support. (*+2)	*Going at the speed of a cruise ship, for how long do I go? Because I may not be able to.* (−1) *I am flexible with the people that really want to (…). If the patient works in the morning and wants to stop smoking, we move the appointment to the afternoon and out of the usual times; we try to do that.* (+1)	*It is very hard for me to prescribe the physical activity; it is very hard.* (−2)
Process			
Planning	*Our actions have to be organized, with everyone and directed to the patient.* (0) *I don't know where we are and where we are going; I just don't know. Sometimes we feel like we lose sight of the goal, ‘listen, what do we need to do now?’.* (0)	*Our goal as professionals is to change patient lifestyle habits. If we can agree on that goal and can do it within a timeframe, that’s perfect; but if not, then we will have to set other timelines.* (0) *Since during the assessment time we were getting little out of it, we told ourselves: ‘let’s assess here, in this other room’. That way we get to the patient in a different way and increase the rate of assessment.* (0)	*Ah, ok, later on you tell me how this is done.* (−1) *When you tell the patient, what exercise, then you write it down on a piece of paper: ‘this patient is interested in doing physical exercise, please help him’. If you send them with that, the gym will pay attention and the patient will get to the appropriate person.* *You didn't know this?* *No, no, I didn't know that, that you have to write it down and send the patient out with that.* (−1) *At the beginning, there was talk of making more time available for PVS but I haven’t done it, from the beginning I refused to do that.* (−2)
Engaging Champions	*This is a team effort, totally personal, without any support; we don’t have anyone who can support it. Then, from primary care, we have been figuring out how to do it.* (+1)	*In any case, these data can be useful to check where we are, but I’m hoping it will not become a burden in our work.* (+2) *Our team coordinator is really motivated. I am with the coordinator. I don't know how to put this, but I have been surprised by the beneficial impact of PVS on our patients.* (+2)	NA
Executing	*I have the impression that we are making it better in each of our meetings and that it becomes clearer.* (+1) *You make the patient fill out the survey at the reception area, because if you don't, then he goes home and doesn't come back.* (+1)	*At the beginning, we felt a bit like: damn, it is difficult to introduce changes, and let’s try to do this slowly, making changes at our own pace, bit by bit.* (0) *Me, the goals, the ones I set are not the ones defined by PVS. To me if someone eats an apple when they don't have anything to eat, I know it will not be counted by PVS but it is an achievement from my perspective. (−*1)	*We are so focused on the health problem and we are not accustomed to working through the healthy themes and how to work with them.* (−2) *In recent months, we have been doing much less assessment and this is because we show up as not doing anything. It is a failing of the computer technology; I don't know really, it is really annoying and it makes me angry that our activity is not being recognized.* (−2)
Reflecting & evaluating	*It would be interesting, even if they are very preliminary data, to have an evaluation of the first semester of the pilot and the changes that we have achieved.* (+1) *We have to look at the outcomes so that we can plan on our own, to be able to see if we are having an impact in the future.* (+1)	*I think we need to put the data in context. The data is a start of the reflection. It is to say: ‘hey, how could we increase the number of patients participating?’* (+1) *Sure, but we’re not sure what the “n” (the expected outcome) means, we don´t understand.* (−1)	*I realized that I was advising patients but I wasn't writing it down (…). It means that if you don't write it down, it is as if you’re not doing anything.* (−2) *I don't know how the indicators are created but other information should emerge from all that, right?* (−2)
Organizational tracking	*We are running around to reach a 95% rate; if it is a pilot, why do we have to reach 95%?* (−2) *At times, we had to make decisions so that the program works and that you have been around to just bring up the statistics.* (−2)	*Man, the statistical part and all that, it is really a pain in the neck, a real drag.* (0) *It is very stressful; it doesn't, really, reflect reality.* (−2)	NA

**Table 4:** CFIR constructs not associated with PVS performance

CFIR Constructs	PVS Cases		
	A	B	C
Intervention characteristics			
Relative advantage	*It is really a great project. For a patient’s own doctor, to guide their eating patterns and to encourage a healthy diet to combat their cholesterol; for my doctor, to tell me that I need to eat broccoli to prevent colon cancer, that’s fantastic. It is great if a professional facilitates that conversation.* (+1)	*I am happy about this. About how to work? Obviously, it has made my clinical work better, that’s clear.* (+1)	*Our profession is going in this direction and with all the chronic patients. To have expanded the work to the whole healthy population has been very important; it raises the profile of these three healthy habits.* (+1)
Complexity	*We have to attend all chronic patients, the ones we have had for a long time. We have that plus the home visits.* (−2)	*If this is a new task at work, if we have to do it and not abandon any of the other present tasks, it is just an additional task; another story is if the task can be accomplished or not, that’s something else.* (−1)	*If we don't one thing, we can do another.* (−2)
Outer setting			
Patient needs & resources	*The patient’s schedule, and then the patients tell you that they will do it, that they know how to eat well, or that they know how to exercise, or that they know about quitting tobacco, then they will do it when they can (…). Then you are left with the question: What do I do with this patient?* (−1)	*It is really hard, seeing the economic conditions that many of our patients are facing. If they are a homemaker and go to the grocery store and have spent a bunch of money on fruits and vegetables, to ask if they are eating their oranges* (all nod and laugh), *well, how can you say that to her, for many, there is not enough money*. (+1)	*I think people get scared, scared of being told off.* (−1)
External policy & incentives	*To be attentive to the project as well as the electronic prescription, and your own clinical work, it is exhausting.* (−2)	*The information technology depends on others and it is very slow when you want to make changes.* (−1)	*I don't know if you have the same impressions from other centers involved but the PVS is an additional task that we have compared to other centers because everything is an imposition from senior management.* (−2)
Inner setting			
Structural characteristics	*They are not short of work, nor her or any of the nurses in this center. To be able to make this program work, we need more nursing resources.* (−2)	*I doubt it; with limited resources we cannot make it all work, because there are so many other tasks that need to be accomplished.* (−2)	*The larger centers have a real difficulty in adapting to structural changes.* (−2)
Readiness for implementation: Available resources	*You bring here a scanner that doesn't work, that doesn't read correctly. Then it doesn't serve any purpose.* (−2)	*Barrier you say? For us, the time that is required.* (−1)	*We are just a physician and a nurse, so the assessment requires a tremendous effort.* (−1)
Process			
Engaging: Formally appointed internal implementation leaders	*The coordinator who runs this motivates us; she is all over us so that we achieve the goal.* (+2)	*She is really motivated. She is more involved with this and she is much more motivated.* (+2)	*A responsible peer has been helping me, many times; he has invested his own time and also mine so that I could learn in a more effective way.* (0)

**Fig. 2:**
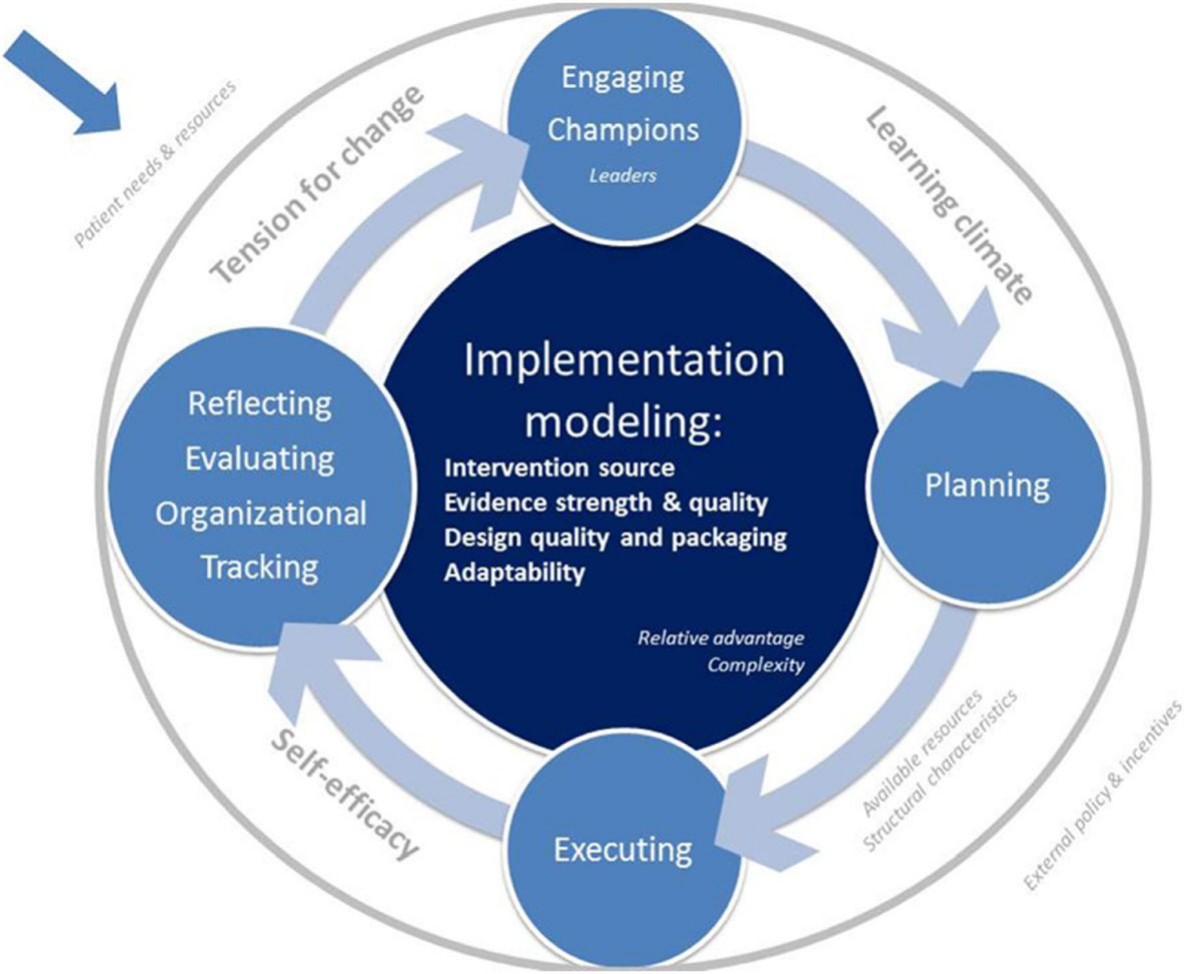
Implementation model for health promotion in primary and community health care

Our emerging conceptual implementation model for health promotion interventions synthesizes the findings. It also highlights the associations of the CFIR constructs with the implementation of innovation in community health and primary care practices (see Fig. 2). Successful implementation appears to be associated with three main components: the context, the implementation process, and the collaborative modelling. We highlight the relationships between these dimensions and potential linkages that require further research. This includes the CFIR constructs that appeared as associated with actual implementation but also others not necessarily associated with performance [15, 19]. Even though the latter were not useful for discriminating between high and low performance, they continue to be important for the implementation model. These variables appear in lowercase in Fig. 2.

#### Context

The organizational conditions associated with implementation performance include *tension for change, learning climate,* and *self-efficacy* [20]. These constructs are interrelated. A positive learning climate nurtures a sense of *self-efficacy* and the development and impact of effective leaders. Similarly, higher feelings of self-efficacy made more resources available to achieve better outcomes. These organizational conditions impact, and yet are influenced by a bottom-up approach to designing and modelling the PVS intervention. The organizational climate is, therefore, the result of the characteristics of individuals, the inner setting, and the overall organizational process, dimensions that compound each other. Overall, the organizational conditions are in dialectical relationship with the context, shaping it as these conditions evolve. Several organizational constructs appear not to be associated with performance. Teams perceive, for instance, the lack of resources as a severe obstacle to change and innovation. Other constructs included in the diagram show a similar pattern. However, these constructs may not be as relevant when making systematic decisions about which teams may be more motivated to change their practices.

#### Implementation process

The constructs *champions, planning, executing,* and *reflecting and evaluating*, are shaped by the aforementioned organizational conditions and generate the implementation collaborative modeling. These local leaders ensure engagement of appropriate team members and community stakeholders and the drawing of resources into the project. In centers where the figure of the local leader is highly valued, the performance was also higher. According to some of the dimensions we have analyzed, a positive set of organizational conditions and practice facilitation provided by the research team shapes successful planning and execution. The ongoing feedback provided by the researchers about the performance of the PHC professional teams plus time for learning-based discussion of these data may produce ambivalent feelings, and PHC centers with the highest implementation rating demonstrated this tension with the highest intensity. In order to avoid negative feelings associated with organizational tracking, the research team should not give feedback to the community teams without first assessing the teams’ own needs and their particular decision-making process.

#### Collaborative modeling

In the PVS first phase, the research team worked collaboratively and transparently with the PHC centers to adapt the 5 A’s intervention (which includes five major steps to develop healthy habits) to the context, guided by action research principles. This engagement included discussions of epidemiological data, community demographics, evidence related to healthy behavior interventions, and the need for an ecological and community approach to prevent and ameliorate chronic illnesses. The degree to which the different teams perceived a need for change varied. However, the introduction of new information and the evolving consensus on needing to change practices may lead highly motivated teams to higher levels of implementation. Without an external research team that helps articulate this process, however, the implementation would stall. The facilitator of this process is the research team but the ownership of the intervention and its characteristics lie in the real local context [21]. The strength of the evidence, therefore, was built on this shared understanding to motivate higher performance among community health teams. The impact of the intervention, the origins of the intervention, the design of support tools and the adaptation to local context nurture the organizational conditions for successful implementation.

## Discussion

Eleven of the thirty-six CFIR constructs are directly related to the level of implementation performance. From the start of the inductive qualitative analysis, we realized that the construct reflecting and evaluating is difficult to assess, since the same PHC professionals valued two distinct aspects during the focus groups. On the one hand, teams which were the most concerned about the implementation process feedback were those with higher levels of implementation effectiveness; in these cases, reflecting and evaluating was positively accepted as an appropriate reflection on action related to how the teams welcome the coaching and feedback was closely connected to the field experience. However, among teams in which the implementation had lower intensity, the impact of the evaluation process is not mentioned. On the other hand, in these cases, reflecting and evaluating generates negative feelings associated with external pressure, and a perception that the external evaluators are judging performance as well as imposing some cumbersome requirements typical of research processes. This reaction appears very strongly in the focus groups. We have reserved a section in Table 2 for this, which is called “organizational tracking”. It is similar to goals and feedback, but we have not categorized it in that way because the evaluation is external to the health center.

Our findings can be compared to those of other studies that have applied the CFIR to evaluate the implementation of health promotion programs in other settings. Similarly to our results, Damschroder and Lowery [15] found that tension for change, learning climate, planning, and reflecting and evaluating clearly distinguish between centers with different effectiveness in the implementation of the MOVE! weight management program in the US Veterans Health Administration PHC centers. These researchers did not find intervention source to be associated with different implementation performance, MOVE! being an externally developed program. In our study, the feeling of ownership of the intervention by the participants emerged as a characteristic strongly associated with implementation performance, and along with evidence strength and quality, strongly distinguished between high and low implementation performance centers. Adaptability and design quality and packaging were not useful for distinguishing between low and high implementation effectiveness of the MOVE! as materials and support tools were consistently considered helpful by all the participating centers. However, in the PVS pilot, a number of failures detected in the new information and communication technology support tools integrated into the electronic health records and delays in fixing them had a negative influence on implementation efforts of the staff of the center with the highest implementation effectiveness, while medium-to-low implementation performance centers were especially appreciative of these support tools. Unlike in the case of the MOVE!, PHC centers piloting PVS designed their own implementation strategy and agreed on well-defined milestones and standard performance measures. Specifically, process constructs were negatively associated with low implementation performance and positively associated with or unaffected by high implementation performance. See Table 4 for quotes that exemplify these constructs differentiating between centers with different levels of implementation performance.

The perception of PHC professionals of the intervention source as internal can be fostered by involving them in discussion, consensus, and a decision making process about priorities in each center according to the specific context and about workflow and the role and contributions of the different members of the PHC professional team. This bottom-up process may lead to a greater sense of ownership and commitment to adhering to the program [22, 23]. Collaborative modeling of the specific implementation strategy for each PHC center in turn favors other constructs associated with the implementation in our study, i.e., *adaptability* and *learning climate*.

In this pilot study, we identified two distinct responses to the monthly provision of clinical performance indicators. One response includes PHC professionals valuing it as a positive contribution because it encourages reflection and prompts team discussions to identify problems and look for solutions. The alternative response is related to the continuous assessment and evaluation, the provision of indicators being perceived as stressful because desired changes depend in part on factors beyond the control of the healthcare providers, i.e., excellence in the design and maintenance of the information technology support tools or requirements imposed by the research protocol associated with the implementation effort. The effectiveness of audit and feedback seems to depend on how the feedback is provided and this requires further investigation [24, 25].

### Limitations

Our research design has limitations inherent to a cross-sectional study of the perception of what explains performance by healthcare professionals, the factors perceived to be associated with positive or negative implementation outcomes being based on the perceptions of the teams’ post facto. The CFIR constructs and performance associations could be bidirectional, are not mutually exclusive categories, and are in continuous evolution. The focus group guide was a set of open-ended questions and was not developed using the CFIR constructs; the CFIR model oriented the analysis only after the initial stage of the grounded consensual qualitative analysis of transcripts. As a result, the lack of data about some of the constructs may be biased by the data collection process rather than a lack of significance of these constructs in the participants’ experience. Our semi-structured data gathering and initial grounded data analysis, however, could have prevented a confirmation bias of the validity of the CFIR framework since the constructs were not employed in the interview design. Further research is required to establish the direction of these associations. To assess for organizational readiness for change, community health centers could be measured via surveys based on a systematic review of applications of the CFIR.

Further, the omission of some of the CFIR constructs may, in some cases, be related to the specific cultural and organizational dynamics of health service delivery in the Basque Country. Other constructs are embedded in or impacted directly by some of the dimensions not mentioned in the groups. For instance, *leadership engagement* is a core aspect of the *learning climate*, a dimension that participants found significant.

## Conclusion

Strong implementation strategies are required to influence the multiple factors associated with innovation in health-promoting practices by PHC professionals. This study identifies a set of factors associated with the implementation of the PVS program (see Fig. 2). In order to develop such strategies, they should be linked to specific actions, techniques, and processes that foster change and tackle barriers [13]. Partnership between clinicians and researchers is required from the design stage. The first step is engaging the majority of the members of the PHC center in a collaborative activity led by PHC professionals appropriately informed about epidemiological and clinical evidence on health promotion interventions and evolving over time through pilot cycles within a learning organization [26].

The findings and recommendations are relevant to primary care practice as it reflects how primary care could be strengthened to highlight the relevancy of prevention measures. In many countries, primary care aims at integrating the preventive effort. Our trials intended to contribute to knowledge related to those assumptions to intentionally include a preventive dimension in family practice. We do believe the paper makes a contribution that bridges implementation and health services research in the context of primary care practice.

## Additional file

Additional file 1: Members of the PVS group. (PDF 193 kb)

## Abbreviations

5 A’s: Ask, Advise, Agree, Assist, and Arrange follow-up
CFIR: Consolidated Framework for Implementation Research
EHR: Electronic health record
PHC: Primary Health Care
PVS: Prescribe Vida Saludable (Prescribe Healthy Life)
UIAPB: The Primary Care Research Unit of Bizkaia
